# Concerning the Cost of Strophanthus

**Published:** 1887-05

**Authors:** 


### MEDICAL NOTES.

### CONCERNING THE COST OF STROPHANTHUS.

Since the new heart-stimulant, strophanthus, has been attracting attention, a charge has been made that a London drug-house had control of the sale of the article, and was charging an excessive price for it.

Messrs. Burroughs, Wellcome & Co., in *The Chemist and Druggist* of March 19th, say:

The original cost of the pods and seeds together—and we shall be pleased to show our books and invoices in evidence—was quite 4*l.* 10*s.* per lb., and we were able to get 4½ oz. of seed out of each pound, so that the seed cost 20*s.* per oz. For a pint of the tincture the following represents the cost, as at first prepared, of 1 in 8 strength:—

| | £ | *s.* | *d.* |
|---|---|---|---|
| Strophanthus seed (2½ oz. at 20*s.* per oz.) | 2 | 10 | 0 |
| Ether, for extracting oil (10 oz.) | | 3 | 0 |
| Spt. vin. rect. (1 pint) | | 2 | 6 |
| Picking, cleaning, and grinding seeds, and other labor | | 1 | 6 |
| Total cost of 1 pint | 2 | 17 | 0 |
| Cost of 1 oz. of tincture would therefore be | | 2 | 10 |
| Glass stoppered bottles (two for each ounce) | | | 5 |
| Wrapping, label, filling, cleaning, etc. | | | 2 |
| Total cost of each ounce in ½-oz. bottles | | 3 | 5 |

Our selling price at first was 2*s.* per ½-oz. bottle, subject to our usual discount to the trade. The cost of distribution in single bottles was very considerable. The above figures, of course, do not take into account the cost of advertising, and the personal efforts and other expenses incidental to procuring and supplying a rare drug like this. The trouble and expense which we have sustained have been very great, and we are dependent upon future sales for any profits on the drug. The difficulties at first experienced in procuring the drug were very great, but we endeavored to distribute strophanthus among the profession and trade as fairly as possible, allowing only one bottle to any one customer; besides this, we distributed a large portion of our stock to hospitals free of charge

In conclusion, we would say that the strophanthus tincture lately sold in New York was prepared from the original lot of the drug which we received. Less than a month ago we reduced the price of the tincture here from 3*s*. to 1*s*. 6*d*. per oz., for we manufactured it from seeds produced at a reduced price.

